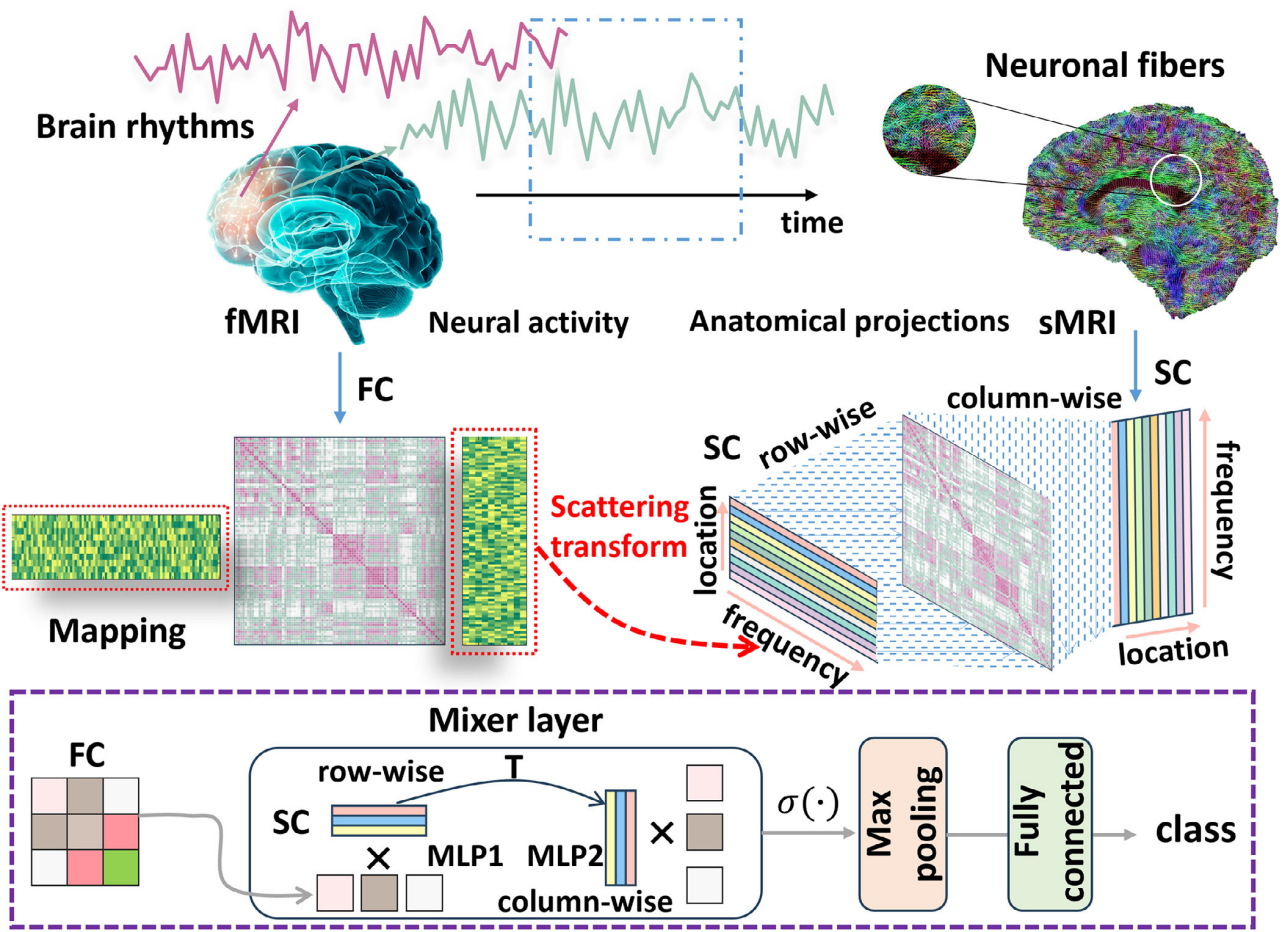# DeepHoloBrain: From Geometric Deep Model to Computational Neuroscience

**DOI:** 10.1002/alz.089911

**Published:** 2025-01-03

**Authors:** Tingting Dan, Guorong Wu

**Affiliations:** ^1^ University of North Carolina, Chapel Hill, NC USA; ^2^ University of North Carolina at Chapel Hill, Chapel Hill, NC USA

## Abstract

**Background:**

The human brain is a complex inter‐wired system that emerges spontaneous functional fluctuations. In spite of tremendous success in the experimental neuroscience field, a system‐level understanding of how brain anatomy supports various neural activities remains elusive.

**Method:**

Capitalizing on the unprecedented amount of neuroimaging data, we present a physics‐informed deep model to uncover the coupling mechanism between brain structure and function through the lens of data geometry that is rooted in the widespread wiring topology of connections between distant brain regions. Since deciphering the puzzle of self‐organized patterns in functional fluctuations is the gateway to understanding the emergence of cognition and behavior, we devise a geometric deep model to uncover manifold mapping functions that characterize the intrinsic feature representations of evolving functional fluctuations on the Riemannian manifold. In lieu of learning unconstrained mapping functions, we introduce a set of graph‐harmonic scattering transforms to impose the brain‐wide geometry on top of manifold mapping functions, which allows us to cast the manifold‐based deep learning into a reminiscent of *MLP‐Mixer* architecture (in computer vision) for Riemannian manifold.

**Result:**

As a proof‐of‐concept approach, we explore a neural‐manifold perspective to understand the relationship between (static) brain structure and (dynamic) function, challenging the prevailing notion in cognitive neuroscience by proposing that neural activities are essentially excited by brain‐wide oscillation waves living on the geometry of human connectomes, instead of being confined to focal areas.

**Conclusion:**

In practice, we evaluate the clinical value of our mixer model in (1) disease early diagnosis and (2) generality of adapting the pre‐trained model to a new dataset. Our extensive experimental results affirm the model’s effectiveness and practicality, marking a significant advancement in the field of neuroscience.